# Clinical Epidemiology and Molecular Investigation of *Protoparvovirus carnivoran1* Infection in Naturally Infected Domestic Cats in Bangladesh

**DOI:** 10.1002/vms3.70480

**Published:** 2025-07-10

**Authors:** Sanjida Ali Sani, Chandan Nath, Md Moktadir Billah Reza, Jannatul Naima, Partha Samanta, Md Saddam Hossain, Md Rayhan Faruque, Md Ahaduzzaman

**Affiliations:** ^1^ Department of Medicine and Surgery Chattogram Veterinary and Animal Sciences University (CVASU) Chattogram Bangladesh

**Keywords:** epidemiology, parvovirus, phylogenetic analysis, *Protoparvovirus carnivoran1*

## Abstract

**Background:**

The infection of cats with *Protoparvovirus carnivoran1* is a global concern due to the likelihood of infection from multiple genetically similar viruses.

**Objectives:**

The present study was undertaken to investigate the clinico‐molecular epidemiology of *Protoparvovirus carnivoran1* infection in naturally infected domestic cats in Bangladesh.

**Methods:**

Rectal swabs (*N* = 100) were collected from cats manifesting clinical signs and screened for the presence of *Protoparvovirus carnivoran1* using polymerase chain reaction (PCR). Selected positive samples (*n* = 38) were partially sequenced for molecular analysis. A structured questionnaire was developed to estimate potential risk factors for *Protoparvovirus carnivoran1* infection, clinical and therapeutic outcomes and overall associations among the variables. Data were analysed using descriptive, univariable and multivariable statistical techniques.

**Results:**

The overall PCR detection rate of the targeted *Protoparvovirus carnivoran1* virus was 99% (99/100). Young and non‐vaccinated animals were mostly infected (*p* < 0.05). The mortality and case fatality of infected cats were 10% and 45%, respectively. The clinical outcomes did not vary between animals receiving different therapeutic groups (*p* = 0.19). The phylogenetic analysis suggests that *Protoparvovirus carnivoran1* isolates share a common ancestor with isolates from different global regions.

**Conclusions:**

The findings indicate that *Protoparvovirus carnivoran1* is circulating in Bangladesh. The clinico‐epidemiological and evolutionary outcomes can be used as a guide for future preventive and control measures against parvovirus infection, both locally and internationally.

## Graphical abstract

The current study reports on the detection patterns of PPVC‐1 in cats in the Chattogram district of Bangladesh during the recent outbreak. The associated risk factors and clinical symptoms specific to the virus are identified. Additionally, the therapeutic outcomes in cats that received different treatment protocols are reported.

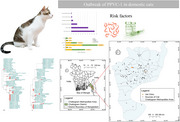

## 1 Introduction

*Protoparvovirus carnivoran1*, formerly known as *Carnivore protoparvovirus1* is a species of the *Protoparvovirus* genus of the family *Parvoviridae*. The *Protoparvovirus carnivoran1* encompasses several phylogenetically similar viruses such as feline parvovirus (FPV), previously known as feline panleukopenia virus (FPLV), and canine parvovirus 2 (CPV‐2; Cotmore et al. 2019; Pénzes et al. 2020). FPV genome comprises approximately 4700 bp, encodes two non‐structural proteins, NS1 and NS2, and two structural proteins, VP1 and VP2 (Liu et al. 2015). While the genome of CPV‐2 is a bit longer, consisting of approximately 5000 bp, it encodes the same two non‐structural proteins, NS1 and NS2, and two structural proteins, VP1 and VP2 (Han et al. 2015). Both viruses are genetically and antigenically closely related, sharing approximately 98% homology. It is believed that CPV‐2 originated from FPV through the acquisition of a few amino acid changes (such as positions 93, 103, 297, 300, 305 and 323) in the VP2 protein, enabling it to cross traverse species barriers (Decaro et al. 2005; Ikeda et al. 2000; Miranda and Thompson 2016; Muz et al. 2012; Ndiana et al. 2021). Soon after it first appeared, CPV‐2 began to evolve, leading to the production of three antigenic variations: CPV‐2a, 2b and 2c (Martella et al. 2006). These variants spread and eventually replaced the original strain of CPV‐2 and are no longer responsible for disease outbreaks since the 1980s (Cavalli et al. 2008). The mutation rate of the VP2 gene is faster and is reported to vary widely across the globe in CPV‐2 than in FPV, which provides advantages to the virus in infecting several carnivores and makes it problematic for CPV‐2 typing based on mutational analysis of the entire genome of the VP2 region (Cavalli et al. 2008; Kaur et al. 2016). Antigenic types may also be detected using real‐time polymerase chain reaction (PCR) tests as a rapid diagnostic method for identifying CPV‐2 variants from rectal swabs of clinically suspected animals (Kaur et al. 2016). Several studies suggest that antigenic classification can be performed by examining the amino acid residue at position 426: 426Asn for CPV‐2a, 426ASP for CPV‐2b and 426Glu for CPV‐2c (Chung et al. 2020; Decaro et al. 2005; Muz et al. 2012). FPV can also be subdivided into three groups based on nucleotide mutations in the VP2 gene: FPV‐G1 (1521A), FPV‐G2 (1521G) and FPV‐G3 (246G, 699C and 1602G) (Karapinar and Timurkan 2024; J. Wang et al. 2024).

Both pathogens caused serious generalised illnesses in cats, predominately characterised by signs of digestive, respiratory, nervous and immune system infection such as fever, anorexia, dullness, diarrhoea, vomiting and dehydration (Abdel‐Baky et al. 2023). Depending on the severity of clinical signs, mortality ranges from 25% to 100% (Pacini et al. 2023; Safwat et al. 2024; Stuetzer and Hartmann 2014). Although vaccination is an effective means of preventing parvovirus, vaccination and immunisation failure still pose challenges for disease eradication (Decaro et al. 2020; Gamoh et al. 2005). Although there is no effective treatment for treating a case of *Protoparvovirus carnivoran1* infection, the random use of antibiotics and symptomatic treatment is a common scenario in many countries, which increases the risk of antibiotic resistance and increases the therapeutic cost. There are several articles on the occurrence (Hasırcıoglu et al. 2023; Ikeda et al. 2000; Pan et al. 2023) and the disease patterns induced by these newly emerged variants of CPV‐2 in cats, or their co‐infections, in different parts of the world. However, there are currently no reports of CPV‐2 occurrence in cats in Bangladesh. Furthermore, there is a lack of information regarding the prevalence of FPV in Bangladesh, and studies are primarily based on symptomatic diagnosis (Chisty et al. 2020), rapid test kit (Islam et al. 2010) or combination of rapid test kit and partial VP2 gene sequence analysis of a single FPV isolate (Chowdhury et al. 2021). The aim of the current study was to investigate the following research questions: (Q1) Are parvovirus outbreaks in cats in Bangladesh caused by classical FPV or multiple variants of CPV‐2 that are causing co‐infection? (Q2) If multiple variants are involved, then does it aggravate the clinical outcomes? (Q3) What are the possible risk factors involved that can be used for future control measures? (Q4) How have the isolates evolved in Bangladesh? And (Q5) is there a need for standardised therapeutic protocols to combat infection?

## 2 Materials and Methods

### 2.1 Study Area and Sample Collection

**FIGURE 1:**
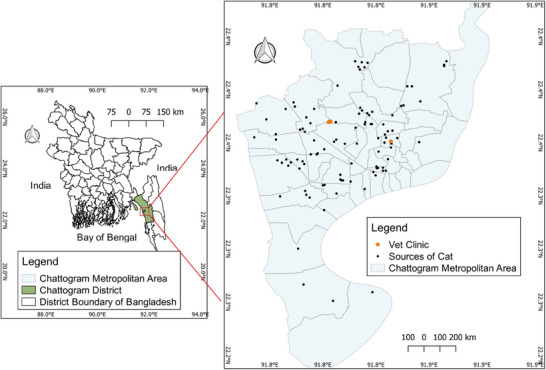
Map of the study area showing the sources of cats transported to veterinary hospitals or clinics for treatment. The map was generated using QGIS v. 2.18.13.

Rectal swab samples from a total of 100 cats with any of these clinical signs of diarrhoea, nasal discharge and vomiting were collected, with the consent of their owners, from three different locations in Chattogram district, Bangladesh, between November 2022 and January 2023 (Figure 1). The majority of the samples were obtained from the University Teaching Veterinary Hospital, which provides outpatient care to approximately 30–40 cats per day from different regions with various clinical conditions. An additional 10 samples were collected from the Veterinary Clinic (*n* = 5) and the Veterinary Hospital (*n* = 5).

### 2.2 Clinical Case Investigation and Data Collection

The cases were identified by the duty veterinarian, and data were recorded according to the case description of this study. Clinical data included rectal temperature (°F), heart rate (bpm/min) and respiration rate (breath/min), degree of dehydration/persistence of skin fold (second), onset of diarrhoea (frequency/day) and onset of vomiting (frequency/day). Descriptive animal data obtained from cat owner included age (< 6/6–12/13–24/> 24 month), body weight (up to 1/> 1–2/> 2–3/> 3 kg), breed (local/Persian/cross), number of cats in the house (1/2–3/> 3), feed source (commercial/homemade/mix), gender (male/female), housing (indoor/feral/semi‐feral), origin of cat (own home/purchased/rescued), routine deworm (yes/no), vaccination against FPV (yes/no) and area location. The animals were followed up to recovery or death. All the animals were treated by the duty doctors based on the symptomatic diagnosis of parvovirus infection. The therapeutic data were retrieved retrospectively from the record book and categorised into three groups: Group 1 (antibiotic + symptomatic + fluid), Group 2 (antibiotic + symptomatic) and Group 3 (symptomatic without antibiotic).

### 2.3 Molecular Screening of *Protoparvovirus carnivoran1*

#### 2.3.1 DNA Extraction

Genomic DNA was extracted from rectal swab samples using a Monarch Genomic DNA Purification Kit (New England BioLabs, USA) according to the manufacturer's instructions. The extracted DNA was stored at −20°C until use.

#### 2.3.2 PCR Procedure

To detect the FPV genome in clinical swab samples, conventional PCR was performed using the primer pair FMF/FMR (3113–3810), which amplifies a 698‐bp fragment of the VP2 gene, as previously described by Mochizuki et al. (1996). To detect the CPV‐2 genome in the same samples, another conventional PCR was conducted using primer pair 555for/555rev (4003–4585), which amplifies a 583‐bp fragment of the VP2 gene as previously described by Buonavoglia et al. (2001).

To test if it was possible to differentiate the CPV‐2a/b/c variants by conventional PCR, three different approaches were used: (a) for CPV‐2a primer pair CPV2aRGM ‘F’/CPV2aRGM ‘R’ (847‐1013), which amplifies a 166‐bp fragment of the VP2 gene of CPV‐2a, as previously described by Kaur et al. (2016) for probe‐based real‐time PCR; (b) for CPV‐2b primer pair Pb sense/Pb anti‐sense, which amplifies a 427‐bp fragment of the VP2 gene of CPV‐2b, as previously described by Pereira et al. (2000) for conventional PCR; and (c) for CPV‐2c primer pair CPV2cRGM ‘F’/CPV2cRGM ‘R’ (1216‐1460), which amplifies a 244‐bp fragment of the VP2 gene of CPV‐2c, as previously described by Kaur et al. (2016) for probe‐based real‐time PCR.

#### 2.3.3 DNA Sequencing and Phylogenetic Analysis

PCR‐positive samples (FMF/FMR = 20, 555for/555rev = 18, CPV2aRGM ‘F’/CPV2aRGM ‘R’ = 6, Pb sense/Pb anti‐sense = 5 and CPV2cRGM ‘F’/CPV2cRGM ‘R’ = 1) of expected length were sent for partial sequencing using the Sanger method to a biotechnology company (Macrogen Inc., South Korea). Both the forward and reverse sequence reads were obtained, and the consensus sequence was created using BioEdit v.7.2 and submitted to NCBI GenBank. Individual sequences were compared with highly similar sequences using the NCBI nucleotide BLAST program (McGinnis and Madden 2004). Selective, highly similar sequences were retrieved with the aim of covering a wider geographical area. The similar sequences combined with the studied sequences were aligned by the ClustalW program of MEGA v.7. Primer set‐specific phylogenetic tree was constructed following nucleotide alignment using ClustalW. The best model was selected based on maximum likelihood fit of nucleotide substitutions considering codon positions 1st, 2nd, 3rd and noncoding sites. For both analyses, the Tamura 3‐parameter model + G was selected based on the best model prediction tool in MEGA V.7. The robustness of the phylogeny was assessed using the bootstrap method with 1000 replicates. Evidence of recombination was evaluated using RDP v.3.44 (Martin 2009).

### 2.4 Statistical Data Analysis

The statistical analyses were performed using JMP Pro v.13. The frequency of PCR‐positive samples was identified using descriptive statistical analysis and presented as a percentage (%). The mortality rate (number of deaths/studied population) and case fatality rate (number of deaths/number of PCR‐positives) were calculated according to a standard formula and presented as percentages (%). The clinical features of PCR‐positive categories were analysed using the standard least squares model due to the normal distribution of data and presented as least square mean ± standard error (LSM±SE). The physiological parameters (rectal temperature, heart rate, respiration rate, degree of dehydration, onset of diarrhoea and onset of vomiting) were assigned as dependent variables, and *Protoparvovirus carnivoran1* PCR‐test results were assigned as independent variables. The significant differences of the tested variable were analysed using the Tukey HSD post hoc test. To identify the potential risk factors, both univariable and multivariable logistic regression analyses were performed. Factors with a *p*‐value ≤ 0.2 from the univariable analysis were taken forward into the multivariable analysis. For multivariable nominal logistic regression analysis, the PCR‐test results of *Protoparvovirus carnivoran1* were assigned as dependent variables, and risk factors selected from univariable analysis were assigned as independent variables. The likelihood ratio test was used to build the model using a backwards elimination approach, where independent variables were chosen for removal based on minimising the log‐likelihood ratio statistics. Additionally, the Wald test was utilised to verify whether the assigned independent variables are collectively significant for the model or not. If the calculated Wald chi‐square (Wald *χ* ^2^) value is greater than the critical value (> 0.05), the coefficient is considered statistically significant, indicating that the predictor has a significant impact on the outcome variable. Otherwise, the coefficient was not considered statistically significant. The clinical outcomes of *Protoparvovirus carnivoran1*‐infected cats treated with three categories of therapeutics were analysed using Pearson's chi‐square test, while the *Protoparvovirus carnivoran1*‐negative cat was excluded during analysis. The classes of antimicrobials used and *Protoparvovirus carnivoran1* outcomes were also analysed in the same way, and additionally, in this analysis, cats that did not receive any antimicrobials (Group 3) were excluded. A significance level of *p* ≤ 0.05 was considered significant throughout.

## 3 Results

### 3.1 Molecular Detection and Evolution of Parvovirus (*Protoparvovirus carnivoran1*) in Cats in Bangladesh

Of the 100 symptomatic cats included in this study, 77 (77%) were PCR‐positive using primer pair FMF/FMR, while 99 (99%) were positive using primer pair 555for/555rev. The NCBI accession numbers were assigned to the partial VP2 genome sequences detected using primer pair FMF/FMR (OR765836–OR765855) and primer pair 555for/555rev (PQ801659–PQ801676). Amino acid residues indicated that all the viruses sequenced using primer pair FMF/FMR (*n* = 20) and primer 555for/555rev (*n* = 18) were FPV (Tables 1 and 2). Since not all PCR‐positive (*n* = 99) samples were sequenced and could potentially include other genogroups like CPV‐2, all positive samples were designated as *Protoparvovirus carnivoran1* positive. Primer pair CPV2aRGM ‘F’/CPV2aRGM ‘R’ (OR785407–OR785412), primer pair Pb sense/Pb anti‐sense (OR785402‐OR785406) and primer pair CPV2cRGM ‘F’/CPV2cRGM ‘R’ (OR785413) successfully amplified the targeted region of VP2 gene of *Protoparvovirus carnivoran1* and were identified as FPV. However, since all the sequenced viruses in this study were found to be FPV, it was not possible to distinguish CPV‐2 strains (a/b/c) based on amino acid residues using these primers. The number of positive samples identified using primer pairs CPV2aRGM ‘F’/CPV2aRGM ‘R’, Pb sense/Pb anti‐sense and CPV2cRGM ‘F’/CPV2cRGM ‘R’ were 99, 74 and 1, respectively (Supporting Information Table S1).

**TABLE 1:** Amino acid variations in the VP2 capsid protein of *Protoparvovirus carnivoran1* among the sequences from this study (OR765836–OR765855) and other GenBank sequences. The sequences analysed in this study were obtained using the PCR product of primer pair FMF/FMR, which yielded an amino acid length of 100–324.

GenBank ID	103	232	267	297	300	301	305	323	324	*Protoparvovirus carnivoran1*
EU498680	V	I	F	S	A	T	D	D	Y	FPV
EU498681	V	I	F	S	A	T	D	D	Y	FPV
MN400979	V	V	F	S	A	T	D	D	Y	FPV
MK052679	V	V	F	S	A	T	D	D	Y	FPV
OR765836	V	V	F	S	A	T	D	D	Y	FPV
OR765837	V	V	F	S	A	T	D	D	Y	FPV
OR765838	V	V	F	S	A	T	D	D	Y	FPV
OR765839	V	V	F	S	A	T	D	D	Y	FPV
OR765840	V	V	F	S	A	T	D	D	Y	FPV
OR765841	V	V	F	S	A	T	D	D	Y	FPV
OR765842	V	V	F	S	A	T	D	D	Y	FPV
OR765843	V	V	F	S	A	T	D	D	Y	FPV
OR765844	V	V	F	S	A	T	D	D	Y	FPV
OR765845	V	V	F	S	A	T	D	D	Y	FPV
OR765846	V	V	F	S	A	T	D	D	Y	FPV
OR765847	V	V	F	S	A	T	D	D	Y	FPV
OR765848	V	V	F	S	A	T	D	D	Y	FPV
OR765849	V	V	F	S	A	T	D	D	Y	FPV
OR765850	V	V	F	S	A	T	D	D	Y	FPV
OR765851	V	V	F	S	A	T	D	D	Y	FPV
OR765852	V	V	F	S	A	T	D	D	Y	FPV
OR765853	V	V	F	S	A	T	D	D	Y	FPV
OR765854	V	V	F	S	A	T	D	D	Y	FPV
OR765855	V	V	F	S	A	T	D	D	Y	FPV
EU659116	A	I	F	S	A	T	Y	N	Y	CPV‐2a
FJ197846	A	I	F	S	A	T	D	N	Y	CPV‐2a
MG763189	A	I	F	S	S	T	D	N	Y	CPV‐2a
AY742932	A	I	F	A	G	T	Y	N	Y	CPV‐2b
AY742934	A	I	F	A	G	T	Y	N	Y	CPV‐2b
MF177258	A	I	F	N	G	T	Y	N	L	CPV‐2b
MK806279	A	I	Y	A	G	T	Y	N	I	CPV‐2c
MH711894	A	I	Y	A	G	T	Y	N	I	CPV‐2c
MF805789	A	I	Y	A	G	T	Y	N	I	CPV‐2c

**TABLE 2:** Amino acid variations in the VP2 capsid protein of *Protoparvovirus carnivoran1* among the sequences from this study (PQ801659–PQ801676) and other GenBank sequences. The sequences analysed in this study were obtained using the PCR product of primer pair 555for/555rev, which yielded an amino acid length of 411–584.

GenBank ID	411	414	415	421	426	445	562	564	567	568	584	*Protoparvovirus carnivoran1*
EU498680	E	W	I	N	N	T	L	N	G	A	Y	FPV
EU498681	E	W	I	N	N	T	L	N	G	A	Y	FPV
MN400979	E	W	I	N	N	T	V	N	G	A	Y	FPV
PQ801659	E	W	I	N	N	T	V	N	G	A	Y	FPV
PQ801660	E	W	I	N	N	T	V	N	G	A	Y	FPV
PQ801661	E	W	I	N	N	T	V	N	G	A	Y	FPV
PQ801662	E	W	I	N	N	T	V	N	G	A	Y	FPV
PQ801663	E	W	I	N	N	T	V	N	G	A	Y	FPV
PQ801664	E	W	I	N	N	T	V	N	G	A	Y	FPV
PQ801665	E	W	I	N	N	T	V	N	G	A	Y	FPV
PQ801666	E	W	I	N	N	T	V	N	G	A	Y	FPV
PQ801667	E	W	I	N	N	T	V	N	G	A	Y	FPV
PQ801668	E	W	I	N	N	T	V	N	G	A	Y	FPV
PQ801669	E	C	N	I	N	T	V	N	G	A	Y	FPV
PQ801670	E	C	N	I	N	T	V	N	G	A	Y	FPV
PQ801671	E	W	I	N	N	T	V	N	G	A	Y	FPV
PQ801672	E	W	I	N	N	T	V	N	G	A	Y	FPV
PQ801673	E	W	I	N	N	T	V	N	G	A	Y	FPV
PQ801674	E	W	I	N	N	T	V	N	G	A	Y	FPV
PQ801675	E	W	I	N	N	T	V	N	G	A	Y	FPV
PQ801676	E	W	I	N	N	T	V	N	G	A	Y	FPV
EU659116	E	W	I	N	N	T	V	S	G	G	Y	CPV‐2a
FJ197846	E	W	I	N	N	T	V	S	G	G	Y	CPV‐2a
MG763189	E	W	I	N	N	T	V	S	G	G	Y	CPV‐2a
AY742932	E	W	I	N	D	T	V	S	G	G	Y	CPV‐2b
AY742934	E	W	I	N	D	T	V	S	G	G	Y	CPV‐2b
MF177258	E	W	I	N	D	T	V	S	G	G	Y	CPV‐2b
MK806279	E	W	I	N	E	T	V	S	G	G	Y	CPV‐2c
MH711894	E	W	I	N	E	T	V	S	G	G	Y	CPV‐2c
MF805789	E	W	I	N	E	T	V	S	G	G	Y	CPV‐2c

**FIGURE 2:**
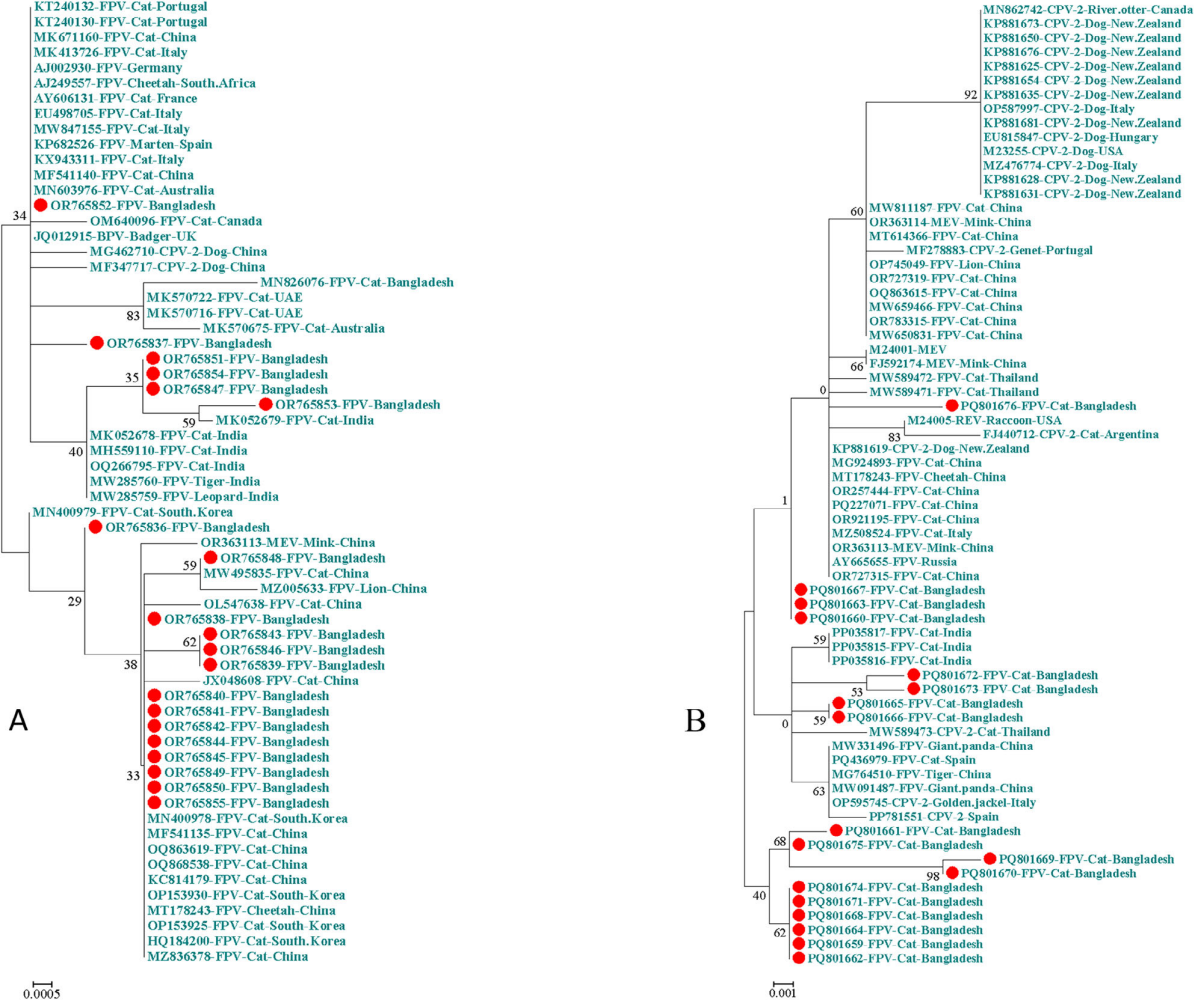
Phylogenetic trees of *Protoparvovirus carnivoran1* utilising primer pair FMF/FMR (A) and primer pair 555for/555rev (B) show a phylogenetic association with similar sequences obtained from GenBank. The isolates in this study are indicated by red dot. The sequences were aligned by ClustalW, and phylogenetic analysis was done by the ML method with 1000 bootstrapping.

The phylogenetic tree of *Protoparvovirus carnivoran1* generated using primer pair FMF/FMR suggests that the viruses (OR765847, OR765851 and OR765853‐54) identified in this study share a common ancestor with a cat FPV (MK052679) from India (Figure 2A). The viruses OR765839, OR765843 and OR765846 formed a distinct clade with low bootstrap support, while the remaining 14 viruses exhibited a polytomy/multiple lineage pattern with isolates from various countries. In contrast, the viruses identified using primer pair 555for/555rev appeared identical and did not show any strong phylogenetic relationship with highly similar GenBank sequences (Figure 2B). FPV identified using primer pair 555for/555rev were classified as FPV‐G1 (1521A). Subdivision of FPV based on primer pair FMF/FMR was not possible, as it sequenced the expected 300–968 nt of VP2 gene of FPV. No evidence of recombination was observed in the analysed viruses based on the VP2 partial gene sequences.

### 3.2 Clinical Outcomes Associated With *Protoparvovirus carnivoran1*infection in Cats

**FIGURE 3:**
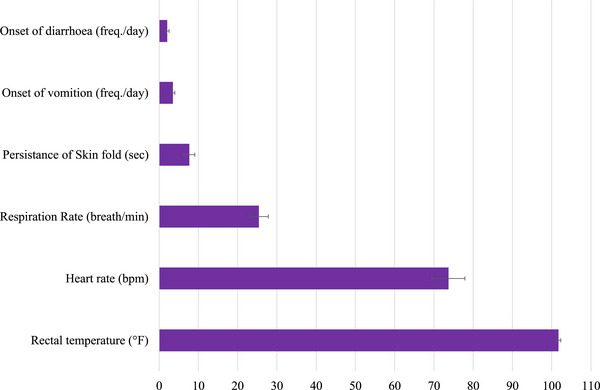
Clinical symptoms exhibited by cats infected with *Protoparvovirus carnivoran1* during 2022–2023 outbreak. Data are presented as least square mean with standard error.

**FIGURE 4:**
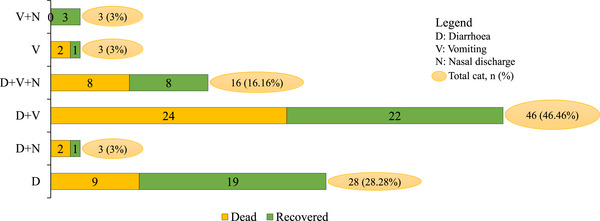
Hallmark symptoms exhibited by *Protoparvovirus carnivoran1*‐infected cats in Bangladesh.

The estimated clinical manifestations associated with *Protoparvovirus carnivoran1* infection in cats are presented in Figure 3. Out of 99 PCR‐positive cats, 45 (45.45%) died, and 54 (54.55%) recovered after 7 days of treatment. The mortality rate and case fatality rate of the infected cats were 10% and 45% respectively. The holistic symptoms in cats tested positive for *Protoparvovirus carnivoran1* are presented in Figure 4.

### 3.3 Risk Factors Associated With *Protoparvovirus carnivoran1*infection in Cats

Ten potential risk factors were considered for univariable analysis, and the results are shown in Table 3. Young animals (mainly ˂ 6 months old) were mostly infected by *Protoparvovirus carnivoran1*. A lack of vaccination against FPV was associated with a high risk of *Protoparvovirus carnivoran1* infection (*p* = 0.003).

**TABLE 3:** Univariate risk factor analysis of *Protoparvovirus carnivoran1* infection in naturally infected domestic cats in Bangladesh. The total significant difference from the overall mean is indicated by bolding.

			*Protoparvovirus carnivoran1*	
Parameter	Category	Population	Pos. (%)	Neg. (%)
Age (month)	< 6	49	**49 (49.49)**	0 (0)
	6–12	32	**32 (32.32)**	0 (0)
	13–24	16	**16 (16.16)**	0 (0)
	> 24	3	2 (2.02)	1 (100)
		*p*‐value	< 0.0001	
Body weight (kg)	Up to 1	12	12 (12.12)	0 (0)
	> 1–2	35	35 (35.35)	0 (0)
	> 2–3	35	34 (34.34)	1 (100)
	> 3	18	18 (18.18)	0 (0)
		*p*‐value	0.60	
Breed	Local	83	82 (82.83)	1 (100)
	Persian	14	14 (14.14)	0 (0)
	Cross	3	3 (3.03%)	0 (0)
		*p*‐value	0.57	
Cat/house	1	30	29 (61.70)	1 (100)
	2–3	10	10 (21.28)	0 (0)
	> 3	8	8 (17.02)	0 (0)
		*p*‐value	0.74	
Feed source	Commercial	9	9 (9.09)	0 (0)
	Homemade	74	73 (73.74)	1 (100)
	Mixed	17	17 (17.17)	0 (0)
		*p*‐value	0.84	
Gender	Male	45	45 (45.45)	0 (0)
	Female	55	54 (54.55)	1 (100)
		*p*‐value	0.36	
Housing	Indoor	57	56 (56.57)	1 (100)
	Feral	1	42 (42.42)	0 (0)
	Semi‐feral	42	1 (1.01)	0 (0)
		*p*‐value	0.68	
Origin of cat	Own home	72	71 (71.72)	1 (100)
	Purchased	17	17 (17.17)	0 (0)
	Rescued	11	11 (11.11)	0 (0)
		*p‐*value	0.82	
Routine deworm	Yes	37	36 (36.36)	1 (100)
	No	63	63 (63.64)	0 (0)
		*p*‐value	0.19	
Vaccination (FPV)	Yes	10	**9 (9.09)**	1 (100)
	No	90	**90 (90.91)**	0 (0)
		*p*‐value	0.003	

Of the different significant risk factors found in the univariable logistic regression analysis of different viruses of *Protoparvovirus carnivoran1* infection, age and vaccination were the most influencing risk factors for *Protoparvovirus carnivoran1* infection (Table 4).

**TABLE 4:** Multivariable nominal logistic regression modelling steps for the investigation of risk factors for FPV and CPV‐2 infection in the 100 cats. The likelihood ratio test (L‐R) was used to build the model using a backwards elimination approach, where independent variables were chosen for removal based on minimising the log‐likelihood ratio statistic.

Regression	Parameter	Category	SE	Wald *χ* ^2^	L‐R	*p*‐value	95% CI		Whole model test	
							Lower	Upper	*χ* ^2^	*p‐*value
Step 1	Age	< 6	2.46	0.0001	6.28	0.0988	−4.84	4.83	11.20	0.0476
		6–12	3.05				−6.00	5.98		
		13–24	3.66				−7.18	7.17		
		> 24	Ref.	—	—	—	—	—		
	RD	Yes	1.81	< 0.000	2.03	0.9999	−3.54	3.55		
		No	Ref.	—	—	—	—	—		
	Vaccination	Yes	1.80	< 0.000	2.77	0.0959	−3.52	3.55		
		No	Ref.	—	—	—	—	—		
										
Step 2	Age	< 6	2.45	0.0001	6.50	0.0896	−3.80	3.75	11.20	0.0244
		6–12	2.99				−4.81	4.80		
		13–24	3.64				−5.86	5.85		
		> 24	Ref.	—	—	—	—	—		
	Vaccination	Yes	1.01	0.0001	3.82	0.0507	0.01	2.77		
		No	Ref.	—	—	—	—	—		

Abbreviations: BW: body weight; RD: routine deworm; L‐R: Likelihood ratio.

### 3.4 Clinical Management of *Protoparvovirus carnivoran1*

There was no significant effect of the therapeutic group on the clinical outcome (death or recovery) of *Protoparvovirus carnivoran1*‐infected cats (Table 5). However, the recovery percentage was comparatively higher in Group 3 cats where no antimicrobials were prescribed. Groups 1 and 2, where various antimicrobial treatment regimens were prescribed, revealed that ceftriaxone was most often used, and antimicrobials from multiple classes were often combined (Table 6). The frequency of *Protoparvovirus carnivoran1* outcomes was similar across the different antimicrobial treatment regimes, so no obvious association between antimicrobial type and *Protoparvovirus carnivoran1* outcomes was found.

**TABLE 5:** The clinical outcomes of *Protoparvovirus carnivoran1*‐infected cats receiving three categories of therapeutics at three veterinary hospitals or clinics in Chattogram, Bangladesh, during 2022–2023's outbreak.

Therapeutic group	Dead (row %)	Recovered (row %)	Total (%)
Group 1	37 (50.0)	37 (50.0)	74 (74.75)
Group 2	4 (44.44)	5 (55.56)	9 (9.09)
Group 3	4 (25)	12 (75.00)	16 (16.16)
Total (column %)	45 (45.00)	55 (55.00)	99 (100)
*p*‐value	0.19		

*Note*: Group 1: antibiotic + symptomatic + fluid, Group 2: antibiotic + symptomatic and Group 3: symptomatic without antibiotic.

**TABLE 6:** Frequency of antimicrobials prescribed to *Protoparvovirus carnivoran1*‐positive 83 cats registered at three veterinary hospitals or clinics in Chattogram, Bangladesh.

Classes of antimicrobials	Antimicrobial	Major symptoms						Outcome (total %)		All (%)
		Diarrhoea		Vomiting		Nasal discharge		Dead	Recovered	
		Yes	No	Yes	No	Yes	No			
Aminopenicillins	Amoxicillin	2	2	4	0	2	2	1 (1.20)	3 (3.61)	4 (4.82)
	Amoxicillin–clavulanic acid	2	0	2	0	0	2	2 (2.41)	0 (0)	2 (2.41)
Aminopenicillins + Nitroimidazole	Amoxicillin–clavulanic acid + Metronidazole	1	0	1	0	0	1	1 (1.20)	0 (0)	1 (1.20)
Cephalosporins	Ceftriaxone	15	17	30	2	8	24	15 (18.07)	17 (20.48)	32 (38.55)
Cephalosporins + Nitroimidazole	Ceftriaxone + Metronidazole	33	3	36	0	10	26	16 (19.28)	20 (24.10)	36 (43.37)
	Ceftiofur + Metronidazole	1	0	1	0	0	1	1 (1.20)	0 (0)	1 (1.20)
Fluoroquinolones	Ciprofloxacin	2	0	2	0	0	2	1 (1.20)	1 (1.20)	2 (2.41)
	Enrofloxacin	1	0	1	0	0	1	1 (1.20)	0 (0)	1 (1.20)
Fluoroquinolones + Nitroimidazole	Ciprofloxacin + Metronidazole	1	0	1	0	0	1	0 (0)	1 (1.20)	1 (1.20)
Nitroimidazole	Metronidazole	3	0	3	0	0	3	3 (3.61)	0 (0)	3 (3.61)
Total								*p* = 0.31		83 (100)
No antibiotic		3	13	16	0	2	14			

## 4 Discussion

This study was intended to solve the research questions related to the aetiology of parvovirus (*Protoparvovirus carnivoran1*) infection in cats in Bangladesh with their clinic‐molecular epidemiology and therapeutics. The study objectives were mostly accomplished by analysing the clinical and molecular data of 100 cats from a recent outbreak in Bangladesh.

The results of the study partially addressed the first research question: Parvovirus (*Protoparvovirus carnivoran1*) outbreaks in Bangladesh appear to be caused by FPV. In this study, 77%–99% of symptomatic cats tested positive for *Protoparvovirus carnivoran1*‐specific PCR, and 38 sequenced samples were identified as FPV, with none testing positive for CPV‐2 (0/38). Previous two studies carried out in Bangladesh to estimate the molecular detection rate of FPV in diarrhoeal cats in Bangladesh found 18.37% (18/98; Chowdhury et al. 2021) and 22.9% PCR‐positive cats in Sylhet, Dhaka, Mymensingh and Rajshahi districts (Kabir et al. 2023). A recent study found a similar 83.3% (25/30) of FPV and 16.6% (5/30) of CPV‐2a/b detection rate in naturally infected cats in Egypt (Magouz et al. 2023). The coinfection was also detected earlier in 24 naturally infected cats in Italy, where the detection rate was 91.6% (22/24) for FPV, 4.2% (1/24) for CPV‐2a and 4.2% (1/24) for CPV‐2c (Battilani et al. 2011). Another study in China found 37.1% (53/143), 0.7% (1/143) and 0.7% (1/143) of cats were co‐infected by FPV, CPV‐2a and CPV‐2c, respectively (Niu et al. 2018). Another study in Turkey found 93.3% (14/15) of cats were positive for FPV, and 6.67% (1/15) were positive for CPV‐2b (Magouz et al. 2023). A study utilised feline samples from Vietnam and Taiwan and revealed that more than 80% of the isolates were of the canine CPV‐2 type rather than FPV (Ikeda et al. 2000). The authors of that study also speculated that the CPV‐2a/2b‐type viruses can spread in cats more efficiently than conventional FPV under natural conditions (Ikeda et al. 2000). Several studies show the emergence of several viruses of *Protoparvovirus carnivoran1* in cats in neighbouring countries of Bangladesh, like India (Mukhopadhyay et al. 2017) and Thailand (Charoenkul et al. 2019). Cats infected with FPV/CPV‐2 variants could potentially be risk factors for spreading infection to other carnivores and the emergence of new variants.

The results of the study did not answer the second research question: whether infection with multiple variants may aggravate clinical outcomes. In this study, no evidence of the presence of CPV variants was detected in the analysis of 38 sequences. However, the possibility cannot be completely ruled out, as all 99 samples were not sequenced due to financial limitations. Although primers developed by Buonavoglia et al. (2001) have been shown to successfully detect CPV‐2 in canine populations (Elbaz et al. 2021), this might not hold true for feline populations. The NCBI Primer‐BLAST program also suggests that both primers can detect different genogroups of *Protoparvovirus carnivoran1* of canine and feline origin. Regarding, CPV‐2 infection in cats, an experimental study investigating the pathogenic potential of CPV types 2a and 2c in domestic cats reported that cats inoculated with CPV‐2a developed symptoms that are similar to the symptoms associated with FPV challenge infection in cats; however, cats inoculated with CPV‐2c also developed the same symptoms that were observed in FPV but in a milder form. Another study found symptoms of fever (103.6°F), anorexia, depression, oral ulcer, gingivitis and haemorrhagic diarrhoea in a cat naturally co‐infected with CPV‐2a and feline calicivirus (Decaro et al. 2010). In another cat of the same study, infected with CPV‐2c presented with a mild form of anorexia, depression and non‐haemorrhagic diarrhoea (Decaro et al. 2010). However, another study reported that cats naturally infected with CPV‐2c can cause severe clinical disease (Miranda et al. 2014). A study reported that CPV‐2b was highly pathogenic in domestic cats (Gamoh et al. 2003). While another study reported that CPV‐2a (Sakamoto et al. 1999) and CPV‐2b (Chalmers et al. 1999) did not generate any overt clinical signs other than leukopenia. Overall, the pathogenicity and clinical outcomes of CPV strains in cats and their co‐infection with FPV are currently very limited to draw an interpretation. Further experimental studies to explore the actual effect of these pathogens in the solo and mixed infection models in feline hosts are required.

The third research question was adequately addressed by the study's findings, which indicated that no immunisation against parvovirus, lack of routine deworming and young age were the main risk factors; hence, these interventions should be taken into account when making future plans to reduce the burden of *Protoparvovirus carnivoran1*. In this study, cats vaccinated against FPV (Biofel PCHR/PureVax Feline 4) were significantly less infected by FPV (3.9%). The findings of this study are supported by another study that reported that vaccination can reduce more than 80% of infections in domestic cats. The authors also found that the vaccine virus‐neutralising antibody titers in the cats experimentally infected with FPV were much lower against CPV‐2c than against FPV (Ikeda et al. 2002). Similar partial protection was also observed in another study for CPV‐2b (Gamoh et al. 2005) and CPV‐2a (Muz et al. 2012). To ascertain whether FPV vaccinations are effective against these more recent antigenic variants, it is imperative that the antigenic character of parvoviruses isolated from domestic cats be continuously monitored. The increase in susceptibility to younger cats was possibly due to a lack of maternally derived antibodies and a weak immune system or to an abundance of mitotically active tissues in the body as supported by earlier reports (Rehme et al. 2022; Stuetzer and Hartmann 2014). Since lack of deworming interferes with nutrition and immune function, anthelminthic treatment was found to be a contributing factor in decreasing the likelihood of CPV‐2 infection in dogs (Miranda et al. 2015). In this study, we did not notice any significant effect of body weight, breed, cat/house, source of feed, gender, housing or the origin of the cat, possibly due to the highly infectious and contagious nature of *Protoparvovirus carnivoran1*.

The answer to the fourth research question was provided: The *Protoparvovirus carnivoran1* sequences that are analysed in this study did not show evidence of recombination events during their evolutionary process and demonstrated ancestral relationships with virus from India. Although the recombination event in *Protoparvovirus carnivoran1* is not uncommon due to its rapid mutation rate and diverse host range (Shackelton et al. 2007), a recombination event between the FPV and CPV‐2 was previously observed in Japan (Ohshima and Mochizuki 2009). Since we did not find evidence of recombination, however, a full VP2 gene or complete genome sequence is required to strengthen the findings. In Bangladesh, like many other resource‐limited countries, the *Protoparvovirus carnivoran1* research is at an early stage, and there are a few related nucleotide sequences available in GenBank. The one FPV isolate identified previously in Bangladesh had a genetic relationship with isolates from the United Arab Emirates (Chowdhury et al. 2021). Additionally, all 18 FPV sequences obtained using primer pair 555for/555rev were identified as FPV‐G1. In has been reported that the prevalence of FPV‐G1 has been increasing in several Asian countries in recent years (Karapinar and Timurkan 2024; R. Wang et al. 2024; Xie et al. 2024).

The answer to the fifth research question would be partially affirmative that there is a need for standardised therapeutic protocols to combat infection according to the findings of this study. Although this study was not designed to assess the different therapeutic protocols through randomised control trial, outcomes were measured based on the hospital record system to understand the field scenario. This observational study showed that use of single or multiple antimicrobials was a common phenomenon irrespective of symptoms; however, it did not affect the recovery rate. This seems to suggest that no systematic approach or antimicrobial selection plan for dealing with *Protoparvovirus carnivoran1* symptomatic infection exists. Apart from this, cats treated with only supportive therapy (analgesic/antiemetic/antihistaminic/fluid) had found relatively higher recovery rate. However, it is important to note that only three cats of this group had diarrhoea, which is a hallmark sign of *Protoparvovirus carnivoran1* infection in cats. Several clinical reviews summarised that supportive therapy and good nursing significantly decrease mortality (Pandey 2022; Rice 2017; Truyen et al. 2009). However, antimicrobials, particularly those that work against gram‐negative and anaerobic bacteria such as aminopenicillins, cephalosporins, aminoglycosides and fluoroquinolones, are the drugs of choice to check secondary bacterial infection (Rice 2017; Truyen et al. 2009), which might be followed by the veterinarian treating cats in this study. The recovery rate was 54.55% in this study, which was higher than another study carried out in Slovakia, where the recovery rate was 11.2% despite the use of immunoglobulins, antibiotics and supportive therapy (Citarová et al. 2022). However, a thorough understanding of the secondary bacteria involved, alteration of gut microbiota and blood biochemical parameters and antimicrobial susceptibility in the particular context of Bangladesh is necessary, which we could not do in this study due to limited funds. With this knowledge, evidence‐based guidelines for treating secondary bacterial infection in *Protoparvovirus carnivoran1* infection could be developed, which could lead to more affordable and effective therapies as well as help prevent human antimicrobial resistance issues. Moreover, it is also equally important to assess the efficacy of antiviral medicine against *Protoparvovirus carnivoran1* infection to reduce the mortality rate; but so far, there is a lack of success (Ferri et al. 2020).

## 5 Conclusion

The FPV was frequently encountered as *Protoparvovirus carnivoran1* pathogen in the recent outbreak in Bangladesh. A symptomatic diagnosis would be possible, however, in the presence of all three hallmark signs. But for confirmation, it is always suggested to adopt molecular tests, particularly in the case of cats presenting a single hallmark sign. The infection was fatal, with a high mortality and case fatality rate, while indiscriminate use of antimicrobials might not be beneficial. A routine vaccination starting at an early stage of life and deworming are equally important for preventing the likelihood of *Protoparvovirus carnivoran1* infection in cats. The chances of the development of a recombinant virus, wider phylogenetic relationships and interspecies transmission capabilities should be continually monitored in order to develop a mass vaccination strategy and design a future control plan.

## Author Contributions

**Sanjida Ali Sani**: investigation, writing—original draft, data curation, validation, funding acquisition. **Chandan Nath**: conceptualisation, investigation, writing—review and editing, software, resources, methodology, validation, visualisation. **Md Moktadir Billah Reza**: investigation, writing—review and editing. **Jannatul Naima**: methodology. **Partha Samanta**: methodology. **Md Saddam Hossain**: methodology. **Md Rayhan Faruque**: supervision. **Md Ahaduzzaman**: conceptualisation, investigation, funding acquisition, writing—original draft, writing—review and editing; visualisation, methodology, formal analysis, software, project administration, supervision, data curation.

## Ethics Statement

Cat owners were informed about the study objectives, and a questionnaire was filled out with their active participation. Rectal swab samples were taken by a registered veterinarian to minimise the discomfort of the animals. Animal handling and therapeutics were provided according to the duties of veterinary doctors. The study design was approved by the authorities of all three hospitals.

## Conflicts of Interest

The authors declare no conflicts of interest.

## Peer Review

The peer review history for this article is available at https://www.webofscience.com/api/gateway/wos/peer‐review/10.1002/vms3.70480.

## Supporting information




**Table S1**: Conventional PCR test results of 100 parvovirus‐suspected cats using different primer pairs targeting various genogroups of *Protoparvovirus carnivoran1*.

## Data Availability

Important datasets that support the conclusions of this article are included within the article and in NCBI GenBank.
