# Supplementary material for: Clinical Epidemiology and Molecular Investigation of Protoparvovirus carnivoran1 Infection in Naturally Infected Domestic Cats in Bangladesh
Source: Vet Med Sci. 2025 Jul 10;11(4):e70480. doi: 10.1002/vms3.70480 (PMC12244267; doi:10.1002/vms3.70480)
Supplement: Supplementary file 1 — Table S1: Conventional PCR test results of 100 parvovirus‐suspected cats using different primer pairs targeting various genogroups of Protoparvovirus carnivoran1. [file VMS3-11-e70480-s001.docx]

**Supplementary table 1.** Conventional PCR test results of 100 parvovirus-suspected cats using different primer pairs targeting various genogroups of *Protoparvovirus carnivoran1*.

| **Sample ID** | **FMF/FMR** | **555for/555rev** | **CPV2aRGM‘F’/CPV2aRGM‘R’** | **Pb sense/Pb anti-sense** | **CPV2cRGM‘F’/CPV2cRGM‘R’** |
| --- | --- | --- | --- | --- | --- |
| 1 | positive | positive | positive | positive | negative |
| 2 | positive | positive | positive | positive | negative |
| 3 | negative | positive | positive | negative | negative |
| 4 | negative | positive | positive | negative | negative |
| 5 | positive | positive | positive | positive | negative |
| 6 | positive | positive | positive | positive | negative |
| 7 | positive | positive | positive | positive | negative |
| 8 | positive | positive | positive | positive | negative |
| 9 | positive | positive | positive | positive | negative |
| 10 | positive | positive | positive | positive | negative |
| 11 | positive | positive | positive | positive | negative |
| 12 | positive | positive | positive | positive | negative |
| 13 | negative | positive | positive | negative | negative |
| 14 | negative | positive | positive | negative | negative |
| 15 | negative | positive | positive | negative | negative |
| 16 | positive | positive | positive | positive | negative |
| 17 | positive | positive | positive | positive | negative |
| 18 | positive | positive | positive | positive | negative |
| 19 | positive | positive | positive | positive | negative |
| 20 | positive | positive | positive | positive | negative |
| 21 | negative | positive | positive | negative | negative |
| 22 | positive | positive | positive | positive | negative |
| 23 | positive | positive | positive | positive | negative |
| 24 | positive | positive | positive | positive | negative |
| 25 | positive | positive | positive | positive | negative |
| 26 | positive | positive | positive | positive | negative |
| 27 | negative | positive | positive | negative | negative |
| 28 | negative | positive | positive | positive | negative |
| 29 | positive | positive | positive | negative | negative |
| 30 | positive | positive | positive | positive | negative |
| 31 | positive | positive | positive | negative | negative |
| 32 | negative | positive | positive | positive | negative |
| 33 | positive | positive | positive | positive | negative |
| 34 | positive | positive | positive | positive | negative |
| 35 | positive | positive | positive | negative | negative |
| 36 | positive | positive | positive | positive | negative |
| 37 | positive | positive | positive | positive | negative |
| 38 | positive | positive | positive | positive | negative |
| 39 | positive | positive | positive | negative | negative |
| 40 | negative | positive | positive | negative | negative |
| 41 | positive | positive | positive | positive | negative |
| 42 | positive | positive | positive | positive | negative |
| 43 | positive | positive | positive | positive | negative |
| 44 | positive | positive | positive | positive | negative |
| 45 | positive | positive | positive | positive | negative |
| 46 | negative | positive | positive | positive | negative |
| 47 | positive | positive | positive | positive | negative |
| 48 | positive | positive | positive | positive | negative |
| 49 | positive | positive | positive | positive | negative |
| 50 | positive | positive | positive | positive | negative |
| 51 | positive | positive | positive | positive | negative |
| 52 | positive | positive | positive | positive | negative |
| 53 | positive | positive | positive | positive | negative |
| 54 | positive | positive | positive | positive | negative |
| 55 | positive | positive | positive | positive | negative |
| 56 | positive | positive | positive | positive | negative |
| 57 | positive | positive | positive | negative | negative |
| 58 | negative | negative | negative | negative | negative |
| 59 | negative | positive | positive | negative | negative |
| 60 | positive | positive | positive | positive | negative |
| 61 | positive | positive | positive | positive | negative |
| 62 | negative | positive | positive | negative | negative |
| 63 | negative | positive | positive | negative | negative |
| 64 | positive | positive | positive | positive | negative |
| 65 | positive | positive | positive | positive | negative |
| 66 | negative | positive | positive | negative | negative |
| 67 | positive | positive | positive | positive | negative |
| 68 | positive | positive | positive | positive | negative |
| 69 | negative | positive | positive | positive | negative |
| 70 | negative | positive | positive | negative | negative |
| 71 | positive | positive | positive | positive | negative |
| 72 | positive | positive | positive | positive | negative |
| 73 | positive | positive | positive | positive | negative |
| 74 | negative | positive | positive | negative | negative |
| 75 | positive | positive | positive | positive | negative |
| 76 | positive | positive | positive | positive | negative |
| 77 | negative | positive | positive | negative | negative |
| 78 | positive | positive | positive | positive | negative |
| 79 | positive | positive | positive | positive | negative |
| 80 | positive | positive | positive | positive | negative |
| 81 | positive | positive | positive | negative | negative |
| 82 | positive | positive | positive | positive | negative |
| 83 | positive | positive | positive | positive | negative |
| 84 | positive | positive | positive | positive | positive |
| 85 | negative | positive | positive | negative | negative |
| 86 | positive | positive | positive | positive | negative |
| 87 | positive | positive | positive | positive | negative |
| 88 | positive | positive | positive | positive | negative |
| 89 | positive | positive | positive | positive | negative |
| 90 | positive | positive | positive | positive | negative |
| 91 | positive | positive | positive | positive | negative |
| 92 | positive | positive | positive | positive | negative |
| 93 | positive | positive | positive | positive | negative |
| 94 | positive | positive | positive | positive | negative |
| 95 | positive | positive | positive | positive | negative |
| 96 | positive | positive | positive | positive | negative |
| 97 | negative | positive | positive | negative | negative |
| 98 | positive | positive | positive | negative | negative |
| 99 | positive | positive | positive | positive | negative |
| 100 | negative | positive | positive | negative | negative |
| Total positive | 77 | 99 | 99 | 74 | 1 |
| Total negative | 23 | 1 | 1 | 26 | 99 |
